# 

**Published:** 1890-03

**Authors:** 


### PERSONAL.

PROFESSOR JAMES MCCALL, principal of the Glasgow Veterinary College, has been added to the editorial staff of *The Veterinarian*, London. This closer union of the Scotch with the English veterinarians will certainly be to the advantage of both and to that of the veterinary profession at large.

### BOOKS AND PAMPHLETS RECEIVED.

"Enucleation of Tuberculous Glands," by Thomas W. Kay, M.D. Reprinted from *Medical Register*.

Bulletin de L'Academie Royale de Medicine de Belgique.

Annales et Bulletin de la Societé de Medicine de Gand.

Bollettino dei Musei di Zoologia ed. Anatomia comparata della R. Universita di Torino. Vol. IV, Nos. 53-73.

Bulletin de la Societé Zoologique de France.

Schriften des Naturwissenschaftlichen Vereins fur Schleswig-Holstein. Band VIII, Heft I.

Bulletin de la Societé Vaudoise der Sciences Naturelles. Lausanne. Vol. XXIV, No. 99.

Johns Hopkins University Circulars. Vol. IX, No. 78.

Canadian Record of Science.

Ornis. Internationale Zeitschrift fur die gesammte Ornithologie. Wein.

Proceedings of the United States National Museum. Vol. XI. Washington, 1889.

Catalogue of Minerals and Synonyms alphabetically arranged for the use of Museums. By T. Egleston, Ph.D. Bulletin U. S. National Museum, No. 330. Washington, 1889.

"The Batrachia of North America." By E. D. Cope. Bulletin U. S. National Museum No. 34. Washington, 1889.

Bibliographical catalogue of the described transformations of North American Lepidoptera. By Henry Edwards. Bulletin U. S National Museum No. 35. Washington, 1889.

A preliminary catalogue of the shell-bearing Marine Mollusks and Bacheopods of the Southeastern coast of the United States, with illustrations of many of the species. By William Healey Hall, A.M. Bulletin U. S. National Museum No. 37. Washington, 1889.

